# Workplace use and outcomes of the dynamic orthosis for lateral epicondylitis: a comparative cohort study

**DOI:** 10.1016/j.jseint.2026.101718

**Published:** 2026-04-30

**Authors:** Kazuhiro Ikeda, Takeshi Ogawa, Shotaro Teruya, Hiromitsu Tsuge, Akira Ikumi, Sho Kohyama, Yuichi Yoshii, Shinzo Onishi

**Affiliations:** a Department of Orthopedic Surgery, Institute of Medicine, University of Tsukuba, Tsukuba, Ibaraki, Japan; b Department of Orthopedic Surgery, Kikkoman General Hospital, Noda, Chiba, Japan; c Department of Orthopaedic Surgery, National Hospital Organization Mito Medical Center, Ibaraki-machi, Higashiibaraki-gun, Ibaraki, Japan; d Department of Orthopedic Surgery, Tokyo Medical University Ibaraki Medical Center, Ami-machi, Inashiki-gun, Ibaraki, Japan

**Keywords:** Lateral epicondylitis, Tennis elbow, Lateral elbow tendinopathy, Orthosis, Orthotic therapy, Work, Presenteeism, Conservative treatment

## Abstract

**Background:**

Orthotic management of lateral epicondylitis remains suboptimal in working patients. Limited symptom relief and poor adherence to brace use during work are the key practical limitations. Therefore, the Dynamic Orthosis for Lateral Epicondylitis (DOLE) was developed for practical use at work. This study aimed to evaluate the work-related effectiveness of DOLE. We compared DOLE with conventional bracing (cock-up wrist splint and counterforce brace) in improving work-related function and pain.

**Methods:**

This retrospective cohort comparison treatment study enrolled employed patients with lateral epicondylitis at a single center. Patients received either conventional bracing (a cock-up wrist splint worn together with a counterforce brace; C group) or DOLE (D group) after its introduction. The primary outcomes were work-related function (Quick Disabilities of the Arm, Shoulder and Hand [QuickDASH] work module) and pain during work (visual analog scale) at 3 and 6 months. Outcomes were compared between groups using analysis of covariance with adjustment for the corresponding baseline value. In addition, at-work brace use was assessed using a questionnaire at 3 months.

**Results:**

Thirty-five patients were included in this study (C group, n = 18; D group, n = 17). At 3 months, at-work use was rare for the cock-up splint (1/18) but common for the counterforce brace (18/18) in the C group. Conversely, 15 of 17 patients in the D group reported wearing DOLE during work. Baseline-adjusted analyses showed significantly better primary outcomes in the D group than in the C group at both 3 and 6 months. At 6 months, the baseline-adjusted between-group differences (D − C) were −23.6 (95% confidence interval, −40.0 to −7.2) for the QuickDASH work module and −23.0 mm (95% confidence interval, −35.0 to −10.9) for work pain on the visual analog scale.

**Conclusion:**

In working patients with lateral epicondylitis, DOLE use was associated with clinically meaningful improvements in the QuickDASH work module and work-related pain compared with conventional bracing. These findings suggest that DOLE may be a feasible work-compatible orthotic option and provide a rationale for larger prospective randomized studies.

Lateral epicondylitis (LE) is a common tendinopathy involving the origin of the extensor carpi radialis brevis (ECRB).^17^^,^^18^^,^^27^, ^28^, ^29^ Its prevalence is reported to be 1%-4%, and it is frequently observed in middle-aged workers performing repetitive upper-limb tasks.^23^^,^^33^^,^^34^ Because LE is attributed to ECRB overuse, treatment focuses on unloading the ECRB origin. Orthoses are a practical means of unloading the ECRB.^23^ Commonly used devices include counterforce braces (tennis elbow braces) and cock-up wrist splints. The counterforce brace compresses the forearm musculature to reduce the tensile load transmitted to the tendon origin.^27^^,^^28^ A cock-up wrist splint immobilizes the wrist, thereby limiting ECRB activation and promoting relative rest at the tendon origin.^10^ Approximately 80% of cases resolve within 6-12 months with nonoperative management.^23^^,^^35^^,^^38^

However, 10%-20% of patients develop persistent symptoms that are refractory to conservative care.^21^^,^^22^^,^^34^ This risk may be higher in working patients because occupational demands often limit opportunities for relative rest.^11^ Workers with limited social support are particularly prone to poor outcomes.^3^ These observations suggest that many workers prioritize maintaining work performance over adhering to treatment recommendations. To address this challenge, orthoses are expected to help workers by facilitating ECRB rest. However, existing devices have limitations in terms of effectiveness and practicality. Counterforce braces provide limited long-term benefits despite short-term increases in pain thresholds.^16^^,^^28^^,^^32^^,^^38^ Although cock-up wrist splints have been shown to provide long-term improvement in some studies,^10^ wrist immobilization can reduce wear compliance among working patients.^1^ Consequently, existing orthotic options do not adequately meet workers' needs to continue working while preserving wrist mobility during treatment. Accordingly, LE imposes a substantial productivity and economic burden.^4^^,^^24^ In refractory cases, patients reportedly require an average of approximately 29 days of sick leave per year,^14^^,^^40^ and work-related losses in the United States have been estimated to exceed USD 22 billion annually.^31^ Preventing chronic LE in workers is therefore a significant societal challenge.

To address these unmet needs, we developed a dynamic orthosis for lateral epicondylitis (DOLE) to enable patients to continue working during treatment (Fig. 1). In developing the DOLE, we specified three design requirements for use at work: (1) preserved wrist mobility, (2) no rigid palmar components that could interfere with gripping, and (3) an easy-to-perform design. DOLE is a wrist-worn device that uses elastic energy to assist the wrist extensor muscles. At rest, the dorsally positioned steel stay maintains the wrist in a functional position. When the wearer flexes the wrist, the stay and neoprene brace elastically deform and store energy directed toward wrist extension. This stored elastic energy dynamically assists wrist extension during motion chains initiated by wrist flexion, such as pickup activities.^16^

**Figure 1:**
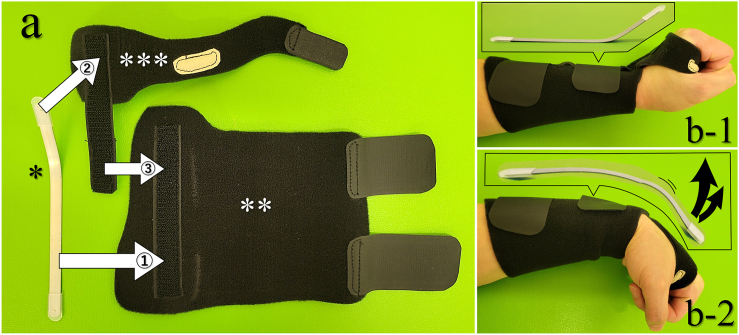
DOLE. (**a**) Components of DOLE. The device consists of a dorsally positioned steel stay (∗) and a neoprene brace that covers the forearm (∗∗) and hand (∗∗∗). The brace has hook-and-loop pockets to house the stay, which are connected by straps (1-3). (**b**) DOLE during wear. At rest, the preset angle of the stay maintains the wrist in an extended (functional) position (**b-1**). With wrist flexion, the stay elastically deforms and stores elastic energy, which is subsequently used to assist wrist extension (**b-2**). *DOLE*, dynamic orthosis for lateral epicondylitis.

This study aimed to assess the feasibility of DOLE for use at work and evaluate its work-related effectiveness. We compared DOLE with conventional bracing (cock-up wrist splint and counterforce brace) in terms of at-work brace use and work-related outcomes.

## Materials and methods

### Study design

The study protocol conformed to the principles of the 1964 Declaration of Helsinki. The institutional review board approved this study (approval no. KC-H26, date: February 5, 2021). Written informed consent was obtained from all participants before enrollment.

This was a nonrandomized, nonconcurrent cohort comparison treatment study conducted at a single center (level of evidence: III). Consecutive eligible patients were managed with conventional bracing during the initial period (C group) and with DOLE after its introduction (D group). The outcomes were predefined and prospectively collected using identical diagnostic criteria, follow-up schedules, and assessment procedures across the two periods.

### Participants

Participants were patients diagnosed with LE between January 2022 and September 2024 at a single secondary care hospital. Eligible patients had clinically meaningful work limitations, defined as a Quick Disabilities of the Arm, Shoulder and Hand (QuickDASH) work module score ≥25 at the initial visit, a threshold informed by prior data in workers with missed work days.^8^ Exclusion criteria were bilateral involvement; concomitant osteoarthritis, collagen vascular disease, or other upper-extremity disorders; and other conditions that could confound work-related outcomes. Patients who discontinued follow-up before the predefined 6-month evaluation while still symptomatic (QuickDASH work module score ≥25) were considered lost to follow-up and were excluded from the final analysis.

**Figure 2:**
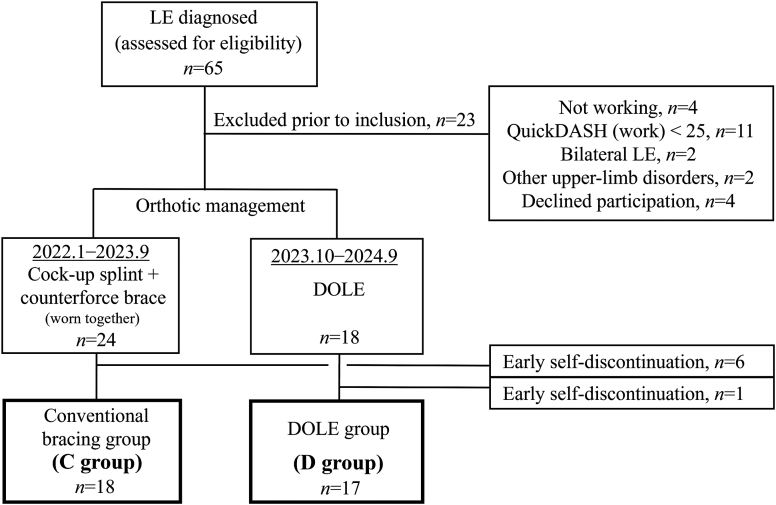
Flow diagram of patient selection. *LE*, lateral epicondylitis; *DOLE*, dynamic orthosis for lateral epicondylitis; *QuickDASH*, Quick Disabilities of the Arm, Shoulder and Hand.

During the study period, 65 patients were diagnosed with LE. Of these, 30 were excluded for the following reasons: bilateral involvement (n = 2), unemployment (n = 4), QuickDASH work module score <25 at the initial visit (n = 11), concomitant medial epicondylitis before treatment (n = 2), early discontinuation of treatment or follow-up (n = 7), or refusal to participate (n = 4). Ultimately, 35 patients (15 men and 20 women; mean age, 53.5 ± 9.4 years) were included in the final analysis (Fig. 2).

### Diagnosis and general management

LE was diagnosed based on localized tenderness over the lateral epicondyle in combination with at least two of the following three findings: a positive Thomsen test, a positive Maudsley test, and lateral elbow pain during gripping.^15^^,^^19^^,^^20^^,^^41^ Plain radiographs of the elbow were obtained from all patients to exclude traumatic or degenerative pathology.

An orthosis was prescribed at the initial visit, and patients were instructed to wear it at work as much as possible. The patients were followed up every 6 weeks over a period of 6 months, and the outcomes were assessed at each visit. At each clinic visit, an occupational therapist provided education on activity modification, forearm muscle relaxation, and orthosis adherence.^25^ Our protocol did not involve oral analgesics or local steroid injections. Patients with a QuickDASH work module score ≥25 at 6 months were classified as having a poor outcome. These patients were followed up for up to 12 months or until their scores fell below 25.

### Orthotic management

**Figure 3:**
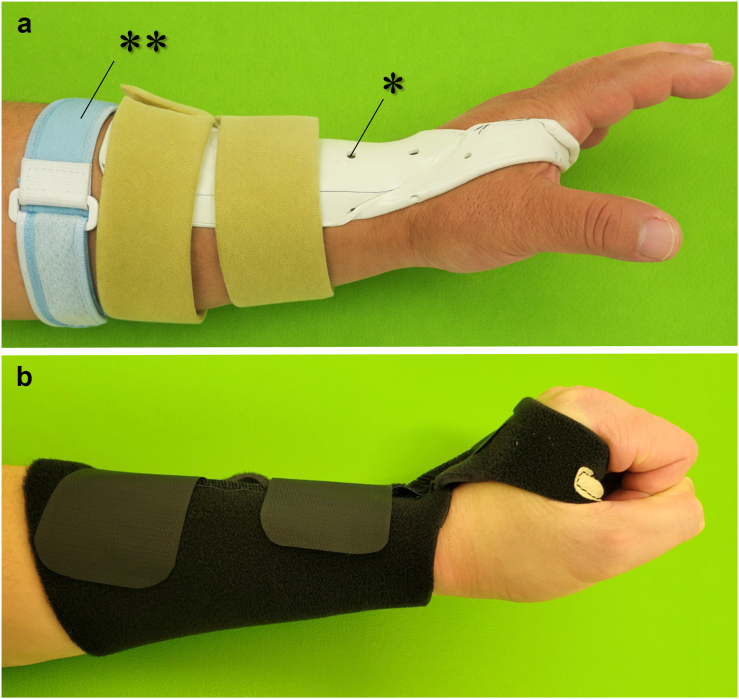
Orthoses used in this study. (**a**) Conventional bracing (C group): combined use of a dorsal cock-up wrist splint (∗) and a counterforce brace (∗∗). The dorsal cock-up splint was custom fabricated by an occupational therapist, set at approximately 30° of wrist extension, and designed with minimal palmar bulk to reduce interference with gripping.^36^ The counterforce was applied at the point of maximal forearm circumference, with the strap tightened as tolerated without skin puckering.^16^^,^^26^ (**b**) DOLE (D group): Patients donned DOLE with the wrist positioned in extension to match the device configuration, and the hand and forearm straps were secured at the maximum tolerated tension without causing discomfort. *DOLE*, dynamic orthosis for lateral epicondylitis.

The details of the bracing protocol are shown in Figure 3. Between January 2022 and September 2023, 18 patients were treated with a combination of a cock-up wrist splint and counterforce brace (conventional bracing; C group). This combination was intended to leverage the complementary strengths of each brace, optimizing both ECRB unloading and at-work wearing compliance.^10^^,^^32^^,^^38^ Between October 2023 and September 2024, 17 patients were treated with DOLE (D group). DOLE was designed to assist equivalent to approximately 50% of the wrist extensor output in healthy adults.^16^^,^^19^^,^^20^ An orthotist fabricated the device using two dorsal stays for men and 1 dorsal stay for women.^16^

### Data collection

The collected data included patient characteristics, at-work orthosis use, and treatment outcomes. The patient characteristics included age, sex, dominant arm involvement, occupation, and symptom duration. Occupation was self-reported and categorized as “mostly sedentary/desk workers” or “manual or physical workers.” Symptom duration was calculated as the number of months from the onset of patient-reported pain to the initial visit. As actual orthosis use at work could influence effectiveness, we assessed at-work use separately from prescriptions. At 3 months, patients completed a questionnaire on at-work brace use, selecting either “wore it most of the time” or “wore it infrequently.” In the C group, the at-work use of the cock-up splint and counterforce brace was assessed separately. The primary outcomes were the QuickDASH work module score and work pain on a visual analog scale (VAS), which was chosen to reflect work-related disability. Secondary outcomes were the QuickDASH disability/symptom score and maximum grip strength ratio (affected/unaffected side).^15^^,^^16^ All outcomes were assessed at three months (early response)^2^ and six months (final follow-up). The rationale for choosing six months as the final assessment is that longer follow-up is often difficult in working patients, and persistent symptoms at six months predict limited further improvement.^35^

To minimize assessor influence, at-work use and patient-reported outcomes (QuickDASH and VAS) were completed outside the examination room and returned to the nonclinical staff. Maximum grip strength was measured using a hand dynamometer (T.K.K.5001; Takei Scientific Instruments Co., Ltd., Niigata, Japan) with the arm at the side, elbow extended, and forearm in neutral rotation.^15^^,^^19^^,^^20^ To minimize discomfort and avoid pain exacerbation that could affect the measurement, grip strength was recorded only once at each visit.^15^^,^^19^^,^^20^

### Statistical analysis

#### Patient characteristics

The patient characteristics were compared between the C and D groups. Normality was assessed using the Shapiro–Wilk test, continuous variables were compared using Student *t* test or the Mann–Whitney *U* test, as appropriate, and categorical variables were compared using the chi-square test.

#### Primary analysis

**Figure 4:**
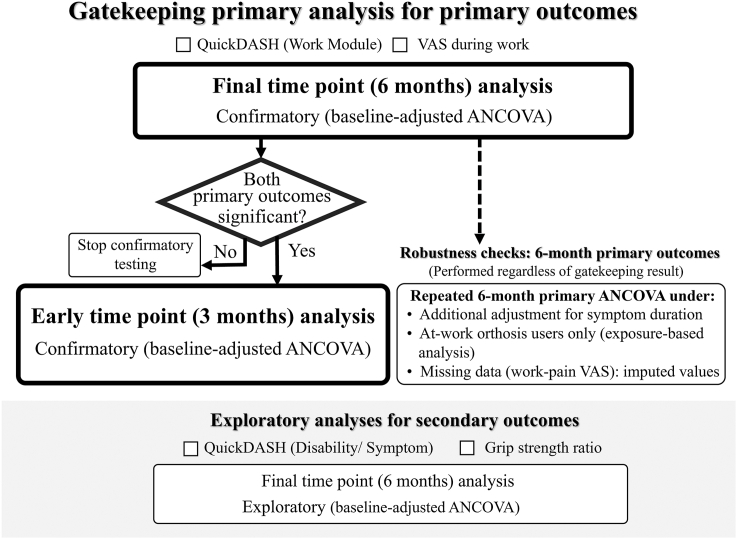
Statistical analysis workflow. *QuickDASH*, Quick Disabilities of the Arm, Shoulder and Hand; *VAS*, visual analog scale; *ANCOVA*, analysis of covariance.

This study used a prespecified gatekeeping strategy for the primary outcomes to control for multiplicity (Fig. 4). We first evaluated between-group differences in both primary outcomes (QuickDASH work module and work-related pain VAS) at 6 months; 3-month between-group comparisons were performed only if both outcomes were statistically significant at 6 months. Between-group comparisons were performed using analysis of covariance (ANCOVA), modeling each follow-up outcome as the dependent variable, with the treatment group and the corresponding baseline value as independent variables. Baseline-adjusted means and adjusted between-group differences with 95% confidence intervals (CIs) were estimated. A group-by-baseline interaction term was tested to assess the homogeneity of the slope assumption; however, the interaction was not significant and was therefore omitted from the final models.

#### Sensitivity analyses

We performed three sensitivity analyses: (1) adding symptom duration to the primary ANCOVA model, (2) an exposure-based analysis stratified by brace use at work, and (3) imputation for missing 6-month work-related pain VAS values (details in the Supplementary Appendix SA). Missing data occurred only for the 6-month work-related pain VAS (four patients in the C group). Accordingly, the primary 6-month VAS analysis used a complete case approach. For the sensitivity analysis, missing values were imputed using a single regression imputation based on a linear model including the group and corresponding baseline value.

#### Exploratory analysis

Secondary outcomes (QuickDASH disability/symptoms and grip strength ratio) were considered exploratory and analyzed using the same ANCOVA approach.

#### Clinical relevance and precision

This study interpreted clinical relevance using previously reported Minimal Clinically Important Differences (MCIDs): 10 points for the QuickDASH (each module) and 10 mm for work-related pain on the VAS.^9^^,^^12^ Because no established MCID exists for the grip strength ratio, we used a measurement-based threshold (0.23) derived from the 95% smallest detectable change for maximum grip strength (see Supplementary Appendix SB).^39^ Within-group and between-group differences were interpreted with reference to these thresholds. No a priori sample size calculation was performed because this was a nonrandomized, nonconcurrent cohort comparison treatment study, and the sample size was limited by the number of eligible patients available during each study phase. Therefore, effect estimates are presented with 95% CIs.

#### General

Continuous variables are presented as means ± standard deviation. Statistical significance was defined as *P* < .05. Analyses were conducted using Bell Curve for Excel (ver. 4.08; Tokyo, Japan).

## Results

### Patient characteristics

The patient characteristics and baseline outcome measures did not differ between the two groups (Table I).

**Table I:** Clinical background of patients.

Group	C group	D group	*P* value
Patient characteristics			
Age	54.8 ± 9.5	52.2 ± 9.6	.508
Sex (woman, %)	66.6	47.1	.241
Manual or physical worker (%)	88.9	94.4	.316
Affected side = dominant hand (%)	83.3	70.6	.369
Duration of illness (mo)	4.6 ± 9.1	3.6 ± 3.4	.741
Baseline outcome measures∗			
QuickDASH score (work)	49.3 ± 26.8	50.7 ± 24.0	.813
Work-related pain on the VAS	58.4 ± 13.7	64.5 ± 20.8	.246
QuickDASH score (disability/symptom)	40.9 ± 17.5	35.3 ± 15.0	.306
Grip strength ratio (affected/unaffected side)	0.61 ± 0.29	0.65 ± 0.25	.609

*C group*, conventional bracing group; *D group*, dynamic orthosis for lateral epicondylitis group; *QuickDASH*, Quick Disabilities of the Arm, Shoulder and Hand; *VAS*, visual analog scale; *ANCOVA*, analysis of covariance.

∗ *P* values are descriptive only; primary analyses were performed using ANCOVA, adjusting for baseline values.

### At-work orthosis use

At 3 months, at-work use in the C group was 1 of 18 (5.6%) for the cock-up splint and 18 of 18 (100%) for the counterforce brace; except for 1 office worker, patients wore only the counterforce brace during work. In the D group, 15 of 17 (88.2%) patients reported wearing the DOLE device during their work. The two patients who did not wear DOLE at work were a manufacturing worker who wore leather gloves and an operating room nurse. Thus, when focusing on actual workplace exposure, the between-group comparison can be interpreted largely as a comparison between the counterforce brace and the DOLE.

### Treatment outcomes over time

**Figure 5:**
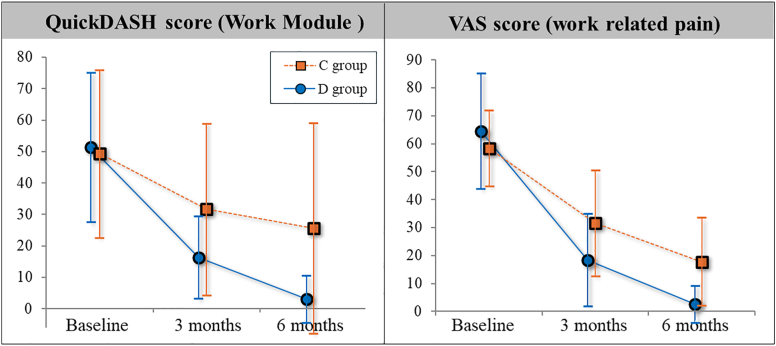
Longitudinal changes in the primary outcomes. Results are presented as mean ± SD. In both groups, both outcomes showed clinically meaningful improvement, exceeding the MCID by 3 months. *QuickDASH*, Quick Disabilities of the Arm, Shoulder, and Hand scores; *VAS*, visual analog scale; *MCID*, Minimal Clinically Important Difference; *SD*, standard deviation.

The longitudinal changes in the primary outcomes (QuickDASH work module and work-related pain on the VAS) are shown in Figure 5, and the descriptive statistics for all outcomes are provided in Supplementary Table S1. By three months, both groups demonstrated clinically meaningful improvements that exceeded the MCID across all outcomes.

### Primary analysis

In the baseline-adjusted ANCOVA, the adjusted between-group differences (D group minus C group) at 6 months favored the D group: −23.6 points for the QuickDASH work module (95% CI, −40.0 to −7.2; *P* = .006) and −23.0 mm for work-related pain on the VAS (95% CI, −35.0 to −10.9; *P* < .001). Both differences were statistically significant and exceeded the MCID, indicating better outcomes in the D group. Because both primary outcomes were significant at 6 months, the primary outcomes at 3 months were evaluated according to the prespecified gatekeeping strategy; the baseline-adjusted between-group differences were statistically significant and exceeded the MCID for both outcomes (Table II). Across all additional sensitivity analyses, the direction and statistical inferences of the between-group differences were unchanged (Supplementary Table S2).

**Table II:** ANCOVA of primary work-related outcomes.

Outcomes	Time points	Adjusted mean		Difference	95% CI	*P* value
		C group	D group			
QuickDASH score (work)	3	31.8	15.6	**−16.2**	−30.4 to −2.0	.026
	6	25.9	2.3	**−23.6**	−40.0 to −7.2	.006
Work-related pain on the VAS	3	30.7	17.2	**−13.5**	−26.3 to −0.8	.039
	6	22.6	−0.4	**−23.0**	−35.0 to −10.9	<.001

*C group*, conventional bracing group; *D group*, dynamic orthosis for lateral epicondylitis group; *QuickDASH*, Quick Disabilities of the Arm, Shoulder and Hand; *VAS*, visual analog scale; *CI*, confidence interval.

Bold values indicate between-group differences exceeding the minimal clinically important difference. Underlined *P* values indicate statistical significance (*P* < .05).

Time points are expressed in months. Adjusted means were estimated using ANCOVA, with baseline values as covariates.

The between-group difference was expressed as the D group minus the C group.

### Exploratory analyses

Baseline-adjusted ANCOVA showed a greater improvement in the QuickDASH disability/symptom score in the DOLE group at 6 months (adjusted mean difference, −11.7; 95% CI, −20.7 to −2.7; *P* = .013), exceeding the MCID. No between-group difference was observed in grip strength ratio (affected/unaffected) at 6 months (Table III).

**Table III:** ANCOVA of secondary outcomes at 6 months.

Outcomes	Adjusted mean		Difference	95% CI	*P* value
	C group	D group			
QuickDASH score (disability/symptom)	16.1	4.4	**−11.7**	−20.7 to −2.7	.013
Grip strength ratio (affected/unaffected side)	0.98	1.06	**+0.09**	−0.00 to 0.17	.056

*ANCOVA*, analysis of covariance; *C group*, conventional bracing group; *D group*, dynamic orthosis for the lateral epicondylitis group; *QuickDASH*, Quick Disability of the Arm, Shoulder and Hand; *CI*, confidence interval.

Bold values indicate between-group differences exceeding the minimal clinically important difference. Underlined *P* values indicate statistical significance (*P* < .05).

Adjusted means were estimated using ANCOVA, with baseline values as covariates.

The between-group difference was expressed as the D group minus the C group.

### Follow-up of poor-outcome cases

In the C group, 8 patients (44.4%) met the poor-outcome criterion at 6 months (QuickDASH work module ≥25); none reported wearing the cock-up splint during work. In the D group, 1 patient (5.9%) met the poor outcome criterion at 6 months and reported not wearing the DOLE device during work. The follow-up and subsequent management of these patients are summarized in Supplementary Table S3.

## Discussion

The primary finding of this study was that DOLE use was associated with better work-related function and lower pain during work than conventional bracing. These benefits were evident as early as three months and were sustained for six months. Consistent with the early improvement in work-related function, the D group showed greater improvement in the QuickDASH disability/symptom score than the C group, indicating concomitant improvement in overall symptoms and function. DOLE enables working patients to pursue treatment while maintaining their work-related functions. To contextualize these effects, we assessed brace-wearing compliance at work. The cock-up splint was seldom worn during work, possibly because wrist immobilization can reduce work efficiency and task speed.^5^^,^^13^^,^^36^^,^^37^ In contrast, at-work compliance with the DOLE and counterforce braces was high. Given these wear patterns, the between-group differences in the primary work-related outcomes likely reflect differences in the dynamic unloading between the DOLE and counterforce brace. Previous studies have shown that dynamic orthoses reduce activity-related pain more than a counterforce brace.^16^ This advantage aligns with biomechanical evidence showing that dynamic orthoses reduce ECRB activity in patients with LE.^7^ Accordingly, the favorable outcomes in the D group may reflect both substantial unloading of the ECRB origin and high at-work compliance. Overall, LE treatment in working patients requires an orthosis that is both effective and wearable.

From the perspective of at-work wearability, previously described dynamic orthoses rely on rigid materials and/or complex constructions.^6^^,^^7^^,^^30^ These features can limit usability at work and create implementation barriers related to comfort, wearability, and supply.^37^ By contrast, DOLE achieves the therapeutic function of a dynamic orthosis with a simple, flexible design for workplace use. This simplified design may reduce the manufacturing complexity and costs, thereby facilitating scalability and broader dissemination. These characteristics support sustainable approaches that maintain work-compatible treatment options for this highly prevalent condition.

This study had several limitations. First, this was a nonrandomized, nonconcurrent cohort comparison treatment study, which may have introduced temporal bias as well as differences over time in clinical practice patterns, patient expectations, and patient education. Because the sample size in this exploratory study was limited by the number of eligible patients available during each study phase, a beta error may have occurred for outcomes without significant between-group differences, such as the affected-to-unaffected grip strength ratio. Second, the outcomes and at-work brace use were self-reported, which may have introduced reporting bias, recall bias, and expectation bias, particularly in this nonblinded study. In addition, objective compliance tracking was not performed. Third, this was a single-center study conducted only in working patients and evaluated only one specific orthosis design (DOLE); therefore, the findings may not be generalizable to non-working patients or to other orthoses with different designs or mechanical properties. Finally, long-term outcomes and societal endpoints such as work productivity were not assessed. Future studies should evaluate DOLE in larger, ideally randomized studies that include objective work-related endpoints (eg, presenteeism).

## Conclusion

In working patients with LE, DOLE use was associated with clinically meaningful improvements in the QuickDASH work module and work-related pain compared with conventional bracing. In addition, the DOLE group showed greater improvement in the QuickDASH disability/symptom score at 6 months. These findings suggest that DOLE may be a feasible work-compatible orthotic option for this population and provide a rationale for larger prospective randomized studies.

## Disclaimers

Funding: This work was supported by 10.13039/501100001691JSPS KAKENHI (Grant Number JP24K23521).

Conflicts of interest: K.I. reports holding Japanese Patent No. 7645603 related to the dynamic orthosis evaluated in this study. Any additional authors, their immediate families, and any research foundations with which they are affiliated have not received any financial payments or other benefits from any commercial entity related to the subject of this article.

## Supplementary data

Supplementary Appendix SA

Supplementary Appendix SB
