# Supplementary material for: Workplace use and outcomes of the dynamic orthosis for lateral epicondylitis: a comparative cohort study
Source: JSES Int. 2026 Apr 30;10(4):101718. doi: 10.1016/j.jseint.2026.101718 (PMC13266164; doi:10.1016/j.jseint.2026.101718)
Supplement: Supplementary Appendix SA [file mmc1.docx]

Appendix A. Measurement-based threshold for the grip strength ratio

Rationale

No established minimal clinically important difference (MCID) has been reported in the grip strength ratio (affected/unaffected side) in patients with lateral epicondylitis. Therefore, we defined a measurement-based threshold by converting the smallest detectable change at the 95% level (SDC_95) of the maximum grip strength reported previously into a ratio.

Source values

In a study by Sveinall et al., the SDC_95 for maximum grip strength was 8.4 kg, and the mean maximum grip strength of the unaffected side was 36.4 kg.39

Derivation

We converted the absolute threshold (kg) into a ratio by dividing it by the mean maximum grip strength of the unaffected side as follows:

Threshold (ratio) = 8.4 / 36.4 = 0.23.

Accordingly, a change in the grip strength ratio of ≥ 0.23 was used as the measurement-based threshold in this study.
