# Supplementary material for: Workplace use and outcomes of the dynamic orthosis for lateral epicondylitis: a comparative cohort study
Source: JSES Int. 2026 Apr 30;10(4):101718. doi: 10.1016/j.jseint.2026.101718 (PMC13266164; doi:10.1016/j.jseint.2026.101718)
Supplement: Supplementary Appendix SB [file mmc2.docx]

Appendix B. Additional analyses for robustness of the primary findings

We conducted additional analyses to examine the robustness of the primary findings.

(i) Symptom duration, a well-established prognostic factor22,34, was added to the primary model (group + baseline value) for sensitivity analysis.

(ii) Based on the compliance survey, we performed an exposure-based analysis restricted to patients who reported wearing the counterforce brace but not the wrist splint during work (C group) and those who wore the DOLE device during work (D group). For both analyses, adjusted between-group differences in primary outcomes were estimated using a baseline-adjusted ANCOVA.

(iii) Missing data were limited to the work-related pain VAS score at 6 months in four patients in the C group. To assess the potential impact of missingness, we performed a single regression imputation using a linear model including group and baseline values, and confirmed the main findings using a complete-case analysis.

These results were consistent with the primary analysis.
