# Supplementary material for: Workplace use and outcomes of the dynamic orthosis for lateral epicondylitis: a comparative cohort study
Source: JSES Int. 2026 Apr 30;10(4):101718. doi: 10.1016/j.jseint.2026.101718 (PMC13266164; doi:10.1016/j.jseint.2026.101718)
Supplement: Supplementary Table S1 [file mmc3.docx]

| Outcomes | Group | Time points | | |
| --- | --- | --- | --- | --- |
|  |  | Baseline | 3 months | 6 months |
| QuickDASH score (Work) | C group | 49.3±26.8 | 31.6±27.6 | 25.7±33.7 |
|  | D group | 50.7±24.0 | 15.8±13.3 | 2.6±7.7 |
|  |  |  |  |  |
| Work-related pain on the VAS | C group | 58.4±13.7 | 31.6±19.2 | 18.8±16.4 |
|  | D group | 64.5±20.8 | 18.5±16.7 | 2.6±6.8 |
|  |  |  |  |  |
| QuickDASH score (Disability/ Symptom) | C group | 40.9±17.5 | 22.0±14.6 | 17.0±17.6 |
|  | D group | 35.3±15.0 | 15.0±9.1 | 4.0±5.1 |
|  |  |  |  |  |
| Grip strength ratio (affected/ unaffected side) | C group | 0.61±0.29 | 0.86±0.20 | 0.97±0.12 |
|  | D group | 0.65±0.25 | 0.95±0.18 | 1.06±0.13 |

Table S1. Outcome measures at baseline, three months, and six months in each group.

C group, conventional bracing group; D group, dynamic orthosis for lateral epicondylitis group; QuickDASH, quick disabilities of the arm, shoulder, and hand scores; VAS, visual analog scale.
