# Supplementary material for: Workplace use and outcomes of the dynamic orthosis for lateral epicondylitis: a comparative cohort study
Source: JSES Int. 2026 Apr 30;10(4):101718. doi: 10.1016/j.jseint.2026.101718 (PMC13266164; doi:10.1016/j.jseint.2026.101718)
Supplement: Supplementary Table S2 [file mmc4.docx]

Table S2. Sensitivity analyses of primary outcomes at six months using baseline-adjusted ANCOVA.

| Analyses | Outcomes | Adjusted mean | | Difference | 95% CI | p value |
| --- | --- | --- | --- | --- | --- | --- |
|  |  | C group | D group |  |  |  |
| ANCOVA of the primary outcomes additionally adjusted for symptom duration | QuickDASH Work Module | 25.1 | 3.2 | **−21.9** | −36.2– −7.5 | 0.004 |
|  | Work-related pain on the VAS | 17.7 | 2.7 | **−15.0** | −25.9– −4.08 | 0.009 |
| ANCOVA results restricted to patients who wore the assigned device at work (counterforce brace or DOLE) | QuickDASH Work Module | 27.2 | 0.8 | **−26.5** | −44.1– −8.9 | 0.005 |
|  | Work-related pain on the VAS | 19.4 | 0.7 | **−18.8** | −29.9– −7.6 | 0.002 |
| ANCOVA of work-related pain on the VAS with imputed missing values | Work-related pain on the VAS | 18.9 | 1.5 | **−17.4** | −28.6 –−6.2 | 0.003 |

The adjusted means were estimated as the overall mean baseline values. Differences represent D group minus C group.

ANCOVA, analysis of covariance; C group, conventional bracing group; D group, dynamic orthosis for lateral epicondylitis group; QuickDASH, quick disabilities of the arm, shoulder, and hand scores; VAS, visual analog scale.
