# Supplementary material for: Workplace use and outcomes of the dynamic orthosis for lateral epicondylitis: a comparative cohort study
Source: JSES Int. 2026 Apr 30;10(4):101718. doi: 10.1016/j.jseint.2026.101718 (PMC13266164; doi:10.1016/j.jseint.2026.101718)
Supplement: Supplementary Table S3 [file mmc5.docx]

Table S3. Follow-up and subsequent management of patients meeting the poor outcome criteria.

| Case ID | At-work orthosis use | QuickDASH Work Module at 6 months | Post–6-month management | Last follow-up  (months) | QuickDASH Work Module at last follow-up |
| --- | --- | --- | --- | --- | --- |
| C-1 | CS: no  CB: yes | 31.3 | Continued conventional orthoses | 15 | 12.5 |
| C-2 | CS: no  CB: yes | 56.3 | Continued conventional orthoses | 9 | 25 |
| C-3 | CS: no  CB: yes | 25 | Switched to DOLE | 12 | 6.3 |
| C-4 | CS: no  CB: yes | 100 | Switched to DOLE | 12 | 0 |
| C-5 | CS: no  CB: yes | 68.8 | Surgery* | 18 | 0 |
| C-6 | CS: no  CB: yes | 25 | Surgery* | 30 | 31.5 |
| C-7 | CS: no  CB: yes | 25 | Discontinued follow-up after 6 months | 6 | 25 |
| C-8 | CS: no  CB: yes | 100 | Discontinued follow-up after 6 months | 6 | 100 |
| D-1 | DOLE: no | 31.3 | Discontinued follow-up after 6 months | 6 | 31.3 |

Poor outcome: QuickDASH Work Module ≥25 at 6 months; C group, conventional bracing group; D group, dynamic orthosis for lateral epicondylitis group; CS, cock-up wrist splint; CB, counterforce brace; DOLE, dynamic orthosis for lateral epicondylitis; QuickDASH, the quick disabilities of the arm, shoulder, and hand scores; *, Surgery was Nirschl procedure.
